# Stability Indicating RP-HPLC Estimation of Atorvastatin Calcium and Amlodipine Besylate in Pharmaceutical Formulations

**DOI:** 10.4103/0250-474X.49117

**Published:** 2008

**Authors:** D. A. Shah, K. K. Bhatt, R. S. Mehta, S. L. Baldania, T. R. Gandhi

**Affiliations:** * Indukaka Ipcowala College of Pharmacy, P. B. No. 53, Vitthal Udyognagar-388 121, India; 1 A. R. College of Pharmacy, P. B. No. 19, Vallabh Vidyanagar-388 120, India; 2 Anand Pharmacy College, Opp. Town Hall, Anand-388 001, India

**Keywords:** Atorvastatin calcium, amlodipine besylate, degradation, reversed phase liquid chromatography, stability indicating, validation

## Abstract

A simple, specific, accurate and stability indicating reversed phase high performance liquid chromatographic method was developed for the simultaneous determination of atorvastatin calcium and amlodipine besylate in tablet dosage forms. A phenomenex Gemini C-18, 5 μm column having 250×4.6 mm i.d. in isocratic mode, with mobile phase containing 0.02 M potassium dihydrogen phosphate:acetonitrile:methanol (30:10:60, v/v/v) adjusted to pH 4 using ortho phosphoric acid was used. The flow rate was 1.0 ml/min and effluents were monitored at 240 nm. The retention times of atorvastatin calcium and amlodipine besylate were 11.6 min and 4.5 min, respectively. The calibration curves were linear in the concentration range of 0.08-20 μg/ml for atorvastatin calcium and 0.1-20 μg/ml for amlodipine besylate. Atorvastatin calcium and amlodipine besylate stock solutions were subjected to acid and alkali hydrolysis, chemical oxidation and dry heat degradation. The degraded product peaks were well resolved from the pure drug peak with significant difference in their retention time values. The proposed method was validated and successfully applied to the estimation of atorvastatin calcium and amlodipine besylate in combined tablet dosage forms.

Atorvastatin calcium (ATV) is chemically 1H-pyrrole-1-heptanoic acid, [R–(R*,R*)]–2-(4-flurophenyl)-β,δ-dihydroxy-5-(1-methylethyl)-3-phenyl-4-[(phenylamino)carbonyl], calcium salt (2:1). Atorvastatin calcium is an inhibitor of 3-hydroxy-3-methyl glutaryl coenzyme A (HMG-Co A) reductase. This enzyme catalyses the conversion of HMG-Co A to mevalonate, an early and rate limiting step in cholesterol biosynthesis^1^–^2^. Amlodipine besylate (AML) is 3-ethyl-5-methyl (4RS)-2-[(2-amino ethoxy) methyl]-4-(2-chlorophenyl)-6-methyl-1,4-dihydropyridine-3,5-dicarboxylate, benzenesulphonate. Amlodipine besylate is a calcium channel blocker. It inhibits the trans membrane influx of calcium ions into vascular smooth muscles and cardiac muscle^3^. The combination dosage form of atorvastatin calcium and amlodipine besylate are available in the market for the treatment of hypertension, chronic stable angina, vasospastic angina, in elevated serum triglyceride levels, primary dysbetalipoproteinemia.

Literature survey revealed that extractive spectrophotometery^4^, liquid chromatographic (LC)^5^–^9^, GC-MS^10^, LC-MS^11^, LC- electrospray tendem mass spectrometry^12^–^14^ and HPTLC^15^ methods have been reported for the estimation of atorvastatin calcium. Amlodipine besylate is official in British Pharmacopoeia^16^. Different LC methods have been reported for the estimation of AML^17^–^21^. For the estimation of amlodipine and atorvastatin combination, spectrophotometeric^22^–^24^, HPTLC^25^ and LC^26^–^29^ methods have been reported. Present study involves development of a stability indicating liquid chromatographic method for the estimation of ATV and AML in combination dosage form.

## MATERIALS AND METHODS

The liquid chromatographic system of Shimadzu make containing LC-10AT (VP series) pump, variable wavelength programmable UV/Vis detector SPD-10AVP and rheodyne injector (7725i) with 20 μl fixed loop was used. Chromatographic analysis was performed using Spinchrom software. A Phenomenex Gemini C18 column with 250×4.6 mm i.d. and 5 μm particle size was used. Analytically pure ATV was procured as gift sample from M/s Blue Cross laboaratory Ltd., (Mumbai, India). AML was procured as gift sample from M/s Torrent pharmaceuticals Ltd (Ahmedabad, India). The purity of ATV and AML were found to be 99.65% and 99.35%, respectively according to the manufacturer's analysis certificates. Methanol, acetonitrile, water (E. Merck, Mumbai, India) was of LC grade, while potassium dihydrogen phosphate and o-phosphoric acid (S.D. fine chemicals, Mumbai, India) were of analytical grade used for the preparation of mobile phase. Tablet formulation A (AVAS-AM, Micro Laboratories Ltd., India) and B (STARCAD, Lupin Labs. Ltd., India) containing labeled amount of 10 mg of atorvastatin and 5 mg of amlodipine were purchased from local market

### Preparation of mobile phase and stock solution:

Potassium dihydrogen phosphate was weighed (0.82 g) and dissolved in 300 ml of water. This solution was mixed with 600 ml of methanol and 100 ml of acetonitrile. The pH was adjusted to 4.0±0.1 using 0.1 M o-phosphoric acid. The solution was filtered, sonicated for 10 minutes for degassing and used as a mobile phase.

Stock solutions were prepared by weighing 25 mg each of AML and ATV and transferring to 2 separate 25 ml volumetric flasks. Volumes were made up to the mark with methanol. The above solutions were further diluted with mobile phase to obtain working standard solutions of 100 μg/ml of each.

### Chromatographic conditions:

A reversed phase C-18 column equilibrated with mobile phase comprising of 0.02 M potassium dihydrogen phosphate: acetonitrile:methanol (30:10:60, v/v/v; pH 4) was used. Mobile phase flow rate was maintained at 1 ml/min and eluent were monitored at 240 nm. A 20 μl of sample was injected using a fixed loop, and the total run time was 15 min. All the chromatographic separations were carried out at controlled room temperature (20-25°).

### Calibration curves for AML and ATV:

Appropriate aliquots of AML and ATV working standard solutions were taken in different 10 ml volumetric flasks and volume was made up to the mark with mobile phase to obtain final concentrations of 0.1, 0.5, 1, 5, 10, 20 μg/ml of AML, 0.08, 0.5, 1, 5, 10, 20 μg/ml of ATV, respectively. The solutions were injected using a 20 μl fixed loop system and chromatograms were recorded. Calibration curves were constructed by plotting peak area versus concentrations of the drug and regression equations were computed for ATV and AML.

### Analysis of Marketed Formulations:

Twenty tablets were weighed accurately and finely powdered. Tablet powder equivalent to 10 mg ATV (and 6.70 mg of AML) was taken in 50 ml volumetric flask. To the above flask, 20 ml of methanol was added and the flask was sonicated for 5 minutes. The solution was filtered in another 50 ml volumetric flask using Whatman filter paper (No.1) and volume was made up to the mark with the same solvent.

Appropriate volume of the aliquot was transferred to a 10 ml volumetric flask and the volume was made up to the mark with the mobile phase to obtain a solution containing 10 μg/ml of ATV and 6.7 μg/ml of AML. The solution was sonicated for 10 min. It was injected as per the above chromatographic conditions and peak area was recorded. The quantifications were carried out by keeping these values to the straight line equation of calibration curve.

### Validation:

The method was validated for accuracy, precision, specificity, detection limit, quantitation limit and robustness. The accuracy of the method was determined by calculating recoveries of AML and ATV by method of standard additions. Known amount of AML (0, 0.5, 5, 20 μg/ml) and ATV (0, 0.5, 5, 20 μg/ml) were added to a pre quantified sample solutions and the amount of AML and ATV were estimated by measuring the peak area and by fitting these values to the straight-line equation of calibration curve.

The instrument precision was evaluated by injecting the solution containing AML (5 μg/ml) and ATV (10 μg/ml) six times repeatedly and peak area was measured. The results are reported in terms of relative standard deviation. The intra-day and inter-day precision study of AML and ATV was carried out by estimating the corresponding responses 3 times on the same day and on 3 different days (first, second and third day) for 3 different concentrations of AML (0.5, 5, 20 μg/ml) and ATV (0.5, 5, 20 μg/ml), and the results are reported in terms of relative standard deviation (RSD). The specificity was estimated by spiking commonly used excipient (starch, talc and magnesium stearate) into a pre weighed quantity of drug. The chromatogram was taken by appropriate dilutions and the quantities of drugs were determined.

The detection limit is defined as the lowest concentration of an analyte that can reliably be differentiated from background levels. Limit of quantification of an individual analytical procedure is the lowest amount of analyte that can be quantitatively determined with suitable precision and accuracy. LOD and LOQ were calculated using following equation as per ICH guidelines. LOD = 3.3 ×σ/S and LOQ = 10 ×σ/S, where σ is the standard deviation of y-intercepts of regression lines and S is the slope of the calibration curve.

Robustness of the method was studied by deliberately changing the experimental conditions like flow rate, percentage of organic phase and also by observing the stability of the sample solution at 25±2° for 24 h. The sample solution was assayed at every 6 h interval up to 24 h.

### Forced degradation study:

Stress degradation study using acid and alkali hydrolysis, chemical oxidation and dry heat degradation was carried out and interference of the degradation products were investigated. ATV and AML were weighed (25 mg each) and transferred to two separate 25 ml volumetric flasks and diluted up to the mark with mobile phase. These stock solutions were used for forced degradation studies.

Forced degradation in basic media was performed by taking 1 ml stock solutions of AML and ATV (1000 μg/ml) in two different 25 ml volumetric flasks and 5 ml of 0.1 N NaOH was added. Similarly, 1 ml aliquots of stock solutions of ATV and AML were taken in same 25 ml volumetric flask and 5 ml 0.1 N NaOH was added. All the flasks were heated in a water bath at 80° for 1 h. After heating solutions were neutralized and diluted up to the mark with mobile phase. Appropriate aliquots were taken from the above solutions and diluted with mobile phase to obtain final concentration of 6 μg/ml of ATV and AML separately and in the mixture. Similarly, forced degradation in acidic medium was performed using 0.1 N HCl.

To perform oxidative stress degradation, appropriate aliquots of stock solutions of ATV and AML (1000 μg/ml) were taken in two different 25 ml volumetric flasks and 5 ml of 3% hydrogen peroxide was added. Similarly, appropriate aliquots of stock solutions of ATV and AML were taken in same 25 ml volumetric flaks and 5 ml 3% hydrogen peroxide was added. All the mixtures were heated in a water bath at 80° for 1 h. After heating all the solutions were diluted with mobile phase to obtain final concentration of 6 μg/ml of ATV and AML separately and in mixture.

To study dry heat degradation, solid drugs were exposed in oven at 80° for 2 h. After 2 h of heating 25 mg each of ATV and AML were weighed and transferred to two separate volumetric flasks (25 ml) and diluted up to the mark with the mobile phase. Solutions were further diluted by taking appropriate aliquots in different 10 ml volumetric flasks to obtain final concentration of 10 μg/ml of ATV and AML. All the reaction solutions were injected in the liquid chromatographic system and chromatograms were recorded.

## RESULTS AND DISCUSSION

**Fig. 1:**
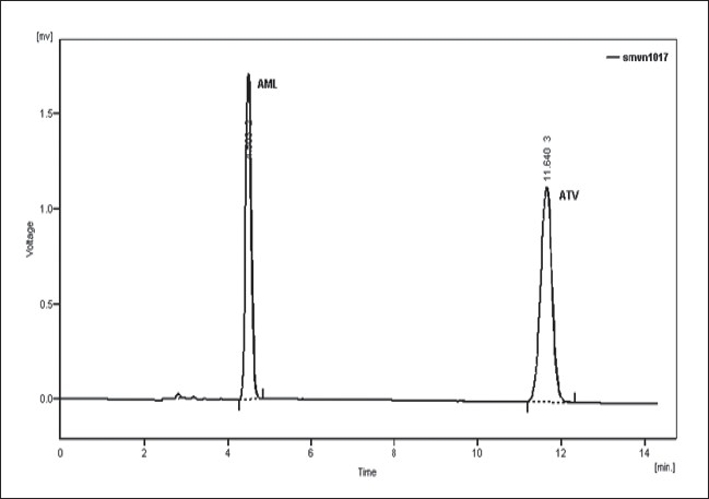
Liquid chromatogram of ATV and AML Liquid chromatogram showing well resolved peaks of ATV (10 μg/ml; 11.6 min) and AML (10 μg/ml; 4.5 min) using mobile phase 0.02 M potassium dihydrogen phosphate:acetonitrile:methanol (30:10:60, v/v/v; pH 4)

**Fig. 2:**
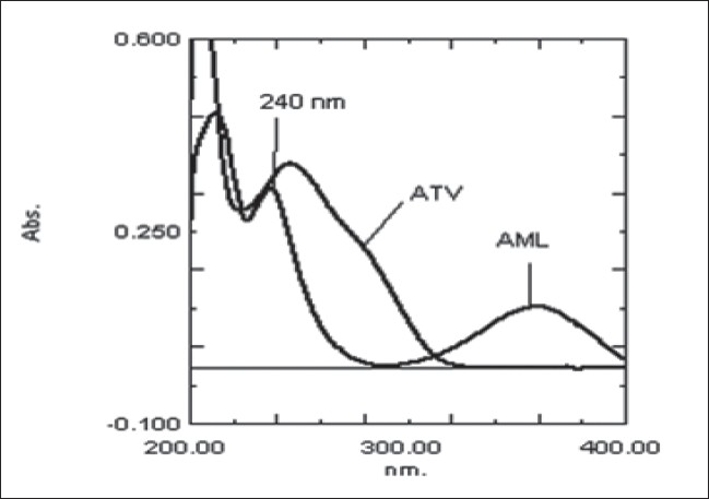
UV overlaid spectra of ATV and AML UV overlaid spectra of ATV (10 μg/ml) and AML (10 μg/ml).

Optimization of mobile phase was performed based on resolution of the drugs and degradation products, asymmetric factor and theoretical plates obtained for AML and ATV. The mobile phase consisting of 0.02 M potassium dihydrogen phosphate: acetonitrile:methanol (30:10:60, v/v/v; pH 4) was selected which gave sharp, well-resolved peaks for AML and ATV (fig. 1). The retention times for AML and ATV were 4.5 and 11.6 min, respectively. The asymmetric factors for AML and ATV were 1.3 and 1.0, respectively. UV overlaid spectra of AML and ATV showed that both the drugs absorbed appreciably at 240 nm, so the same was selected as the detection wavelength during the studies (fig. 2). The calibration curve was found to be linear over the range of 0.1-20 μg/ml for AML and 0.08-20 μg/ml for ATV. The data of regression analysis of the calibration curves are shown in Table 1.

**TABLE 1:** REGRESSION ANALYSIS OF THE CALIBRATION CURVE FOR THE PROPOSED METHOD

Parameters	AML	ATV
Linearity range (μg/ml)	0.1 - 20	0.08 - 20
Slope	24.45	29.66
Standard deviation of slope	0.188	0.828
Intercept	−0.253	−1.16
Standard deviation of intercept	0.0301	0.797
Correlation coefficient	0.9999	0.9998

The accuracy of the method was determined by calculating recoveries of AML and ATV by method of standard additions. The recoveries obtained were 98.73-99.83% for AML and 98.52-99.38% for ATV, respectively. The high values indicate that the method is accurate. Instrument precision was determined by performing injection repeatability test and the RSD values for AML and ATV were found to be 0.53% and 0.36%, respectively. The intra-day and inter-day precision studies were carried out. For the intra-day study RSD values were found to be 0.35-1.07% for AML and 0.58-1.32% for ATV and for inter-day precision study RSD values were found to be 0.36- 1.86% for AML and 0.66-1.74% for ATV, respectively. The low RSD values indicate that the method is precise. The detection limits for AML and ATV were 0.04 μg/ml and 0.03 μg/ml, respectively, while quantitation limits were 0.1 μg/ml and 0.08 μg/ml, respectively. The above data shows that a nanogram quantity of both the drugs can be accurately and precisely determined. The validation parameters are summarized in Table 2 and the system suitability test parameters are shown in Table 3.

**TABLE 2:** SUMMARY OF VALIDATION PARAMETERS

Parameters	AML	ATV
Detection limit (μg/ml)	0.04	0.03
Quantitation limit (μg/ml)	0.1	0.08
Accuracy (%)	98.73-99.83%	98.52-99.38%
Precision (RSD^a^,%)		
Intra-day precision (n=3)	0.35-1.07%	0.58-1.32%
Inter-day precision (n=3)	0.36- 1.86%	0.66- 1.74%
Instrument precision (RSD^a^)	0.53%	0.36%
Robustness	98.37-100.25%	97.45-99.58%

a RSD is relative standard deviation and ‘n’ is number of determinations

**TABLE 3:** SYSTEM SUITABILITY TEST PARAMETERS FOR THE PROPOSED METHOD

System suitability parameters	ATV	AML
Retention time	11.6 min	4.5 min
Theoratical plates/ meter	10130	6856
Assymetric factor	1.0	1.3
Resolution	21	-

Robustness of the method was studied by changing the flow rate of the mobile phase from 1 ml/min to 0.8 ml/min and 1.2 ml/min. Using 1.2 ml/min flow rate, retention time for AML and ATV were observed to be 4.0 and 10.2 min, respectively and with 0.8 ml/min flow rate, retention times for AML and ATV were observed to be 4.9 and 12.8 min, respectively without affecting the resolution of the drugs. When mobile phase composition was changed to 0.02 M potassium dihydrogen phosphate:acetonitrile:methanol (25:15:60, v/v/v; pH 4) by increasing percentage of acetonitrile, the retention time for AML and ATV were observed to be 4.2 and 10.9 min, respectively. The solution stability study revealed that AML and ATV solutions were stable for 24 h without detectable degradation and the percentage recovery of both the drugs were found to be more than 97%.

**Fig. 3:**
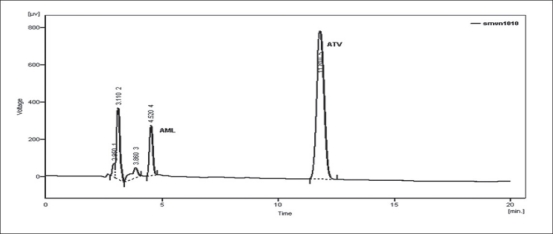
Chromatogram of base treated ATV and AML Chromatogram of 0.1 N NaOH-treated ATV (6 μg/ml) and AML (6 μg/ml) at 80° for 1 h

**Fig. 4:**
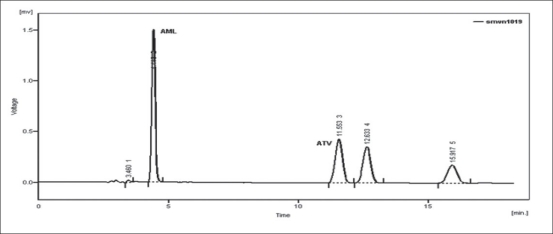
Chromatogram of acid treated ATV and AML Chromatogram of 0.1 N HCl-treated ATV (6 μg/ml) and AML (6 μg/ml) at 80° for 1 h.

**Fig. 5:**
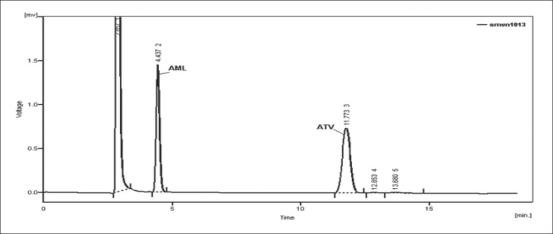
Chromatogram of hydrogen peroxide treated ATV and AML Chromatogram of 3% hydrogen peroxide-treated ATV (6 μg/ml) and AML (6 μg/ml) at 80° for 1 h.

**Fig. 6:**
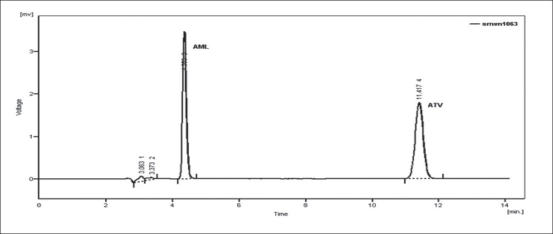
Chromatogram of dry heat degradation study of both the drugs Chromatogram of dry heat degradation study of ATV (10 μg/ml) and AML (10 μg/ml) at 80° for 2 h

Forced degradation study was carried out by subjecting both the drugs to acid and alkali hydrolysis, chemical oxidation and dry heat degradation conditions. The chromatograms of base degraded sample showed degradation product peaks at retention time (RT) 2.95, 3.11 and 3.86 min for AML and ATV was found to be stable to base degradation (fig. 3). The peaks of the degradation products were well resolved from the drug peaks. Acid hydrolysis study showed that AML was stable in acidic condition but ATV gave degradation product peaks at RT 12.63 and 15.92 min (fig. 4). Oxidative stress degradation resulted in degradation product peaks at RT 12.85 and 13.68 min for ATV and AML was indicated to be stable (fig. 5). Dry heat degradation study resulted in to degradation product peaks at 3.06 and 3.37 min for AML and ATV was found to be stable (fig. 6).

The degradation study thereby indicated that AML was stable to acid and chemical oxidation study while it was susceptible to base hydrolysis and dry heat degradation. ATV was indicated to be susceptible to acid hydrolysis and chemical oxidation, with maximum degradation in acid (Table 4). Under above condition, specificity was demonstrated as no degradation products from different stress conditions affected determination of ATV and AML.

**TABLE 4:** FORCED DEGRADATION STUDY OF ATV AND AML FOR THE PROPOSED METHOD

Condition	Time (h)	Recovery (%)		Retention time of degradation products	
			
		ATV	AML	ATV	AML
Base 0.1 N NaOH^b^	1	97.56	16.55	-	2.95, 3.11, 3.86
Acid 0.1 N HCl^b^	1	38.04	96.36	12.63, 15.92	-
3% hydrogen peroxide^b^	1	82.58	97.51	12.85, 13.68	-
Dry heat^b^	2	97.92	94.62	-	3.06, 3.37

b samples were heated at 80° for specified period of time.

The proposed method was applied to the determination of AML and ATV in their combined dosage form (Tablet A and B). The results for AML and ATV were comparable with the corresponding labeled amounts (Table 5). Compared to the reported methods^26^–^29^, the proposed liquid chromatographic method is simple, accurate, specific and degradation products peaks are well resolved from the drug peaks.

**TABLE 5:** ASSAY RESULTS OF TABLET DOSAGE FORM USING PROPOSED METHOD

Formulations	Labelled Amount (mg)		% Recovery^c^	

	ATV	AML	ATV	AML
A	10.34	6.93	98.66±0.68	98.21±0.98
B	10.34	6.93	98.80±1.05	99.90±0.82

c mean±standard deviation of three determinations; Tablet formulation A is AVAS-AM (Micro Laboratories Ltd., India) and B is Starcad (Lupin Labs. Ltd., India) containing labeled amount of 10 mg of atorvastatin and 5 mg of amlodipine.
